# Antibodies targeting *Candida albicans* Als3 and Hyr1 antigens protect neonatal mice from candidiasis

**DOI:** 10.3389/fimmu.2022.925821

**Published:** 2022-07-22

**Authors:** Shakti Singh, Sunna Nabeela, Ashley Barbarino, Ashraf S. Ibrahim, Priya Uppuluri

**Affiliations:** ^1^ Division of Infectious Diseases, The Lundquist Institute for Biomedical Innovation at Harbor, University of California Los Angeles (UCLA) Medical Center, Torrance, CA, United States; ^2^ David Geffen School of Medicine at UCLA, Los Angeles, CA, United States

**Keywords:** neonates, antibodies, Als3, Hyr1, neonatal candidiasis, vaccine

## Abstract

Pre-term infants in neonatal intensive care units are vulnerable to fungal sepsis. In this patient population, *Candida albicans* remains the predominant fungal pathogen causing high morbidity and mortality, despite antifungal therapy. Thus, new preventative/therapeutic strategies against neonatal candidiasis are needed. Previously, we have reported that vaccination with recombinant forms of the *C. albicans* N-termini of the cell wall proteins Als3 (rAls3p-N) and Hyr1 (rHyr1p-N) protected adult mice from disseminated candidiasis. Further, in a Phase 1b/2a NDV-3A (an rAls3p-N formulated with alum) protected women from recurrent vulvovaginal candidiasis, with anti-Als3p IgG2 isotype being a biomarker for efficacy. Here, we performed a proof of concept study to evaluate if anti-Als3p or anti-Hyr1p antibodies are important for prevention of disseminated candidiasis in neonates. Als3 and Hyr1 antigens when adjuvanted with complete Freund’s adjuvant (CFA)/incomplete Freund’s adjuvant (IFA) induced a robust antibody response with a ten-fold higher titer of IgG2, than attained by either antigen formulated with alum. Transplacental transfer of these antibodies significantly reduced fungal burden in the kidneys of mice pups, and adoptive transfer of vaccinated mothers’ sera into pups displayed similar levels of protection. Neutrophils were found important for this efficacy. Finally, anti-Hyr1 antisera potentiated the activity of fluconazole in protecting from *C. albicans* infection. Our current studies are the first in the field to emphasize the importance of anti-Als3 and anti-Hyr1 antibodies in preventing neonatal candidiasis. Considering that *Candida* infections in low birthweight infants is a lethal infection, active and passive vaccination strategies using these antigens could have profound clinical relevance.

## Introduction

Four million neonates are born every year in the United States. About 11.4% are born preterm (between 28 and 36 weeks of gestation), 8% have low birth weight (LBW) and 1.4% are of very low birth weight (VLBW) (1). It is recognized that 75% of infants admitted to the neonatal intensive care unit (NICU) are colonized by *Candida* by the first month (2). Infection is acquired by vertical transmission during vaginal delivery, postnatally from contact with maternal skin or the skin of direct care providers, or direct transmissions *via* contaminated equipment or intravenous catheters. *Candida albicans* remains the most prominent pathogen in neonates, followed by significant cases due to *C. parapsilosis (* 3–5 *). Candida* infections are responsible for ~10-12% of nosocomial sepsis in VLBW (<1500 g) infants, with a collective incidence of up to 4% among all NICU admissions (6). In fact, *Candida* is the 3rd most frequently isolated organism (after coagulase negative *Staphylococcus* spp. and *S. aureus*) in late onset sepsis in VLBW infants (2). Despite empirical antifungal therapy, mortality related to the disease remains considerably high (20-30%), with even higher rates (59-73%) of long-term neurodevelopmental impairment in survivors (2, 6).

Protection against infection in premature neonate depends upon the innate immune system, and on antibodies acquired passively from the mother (7). The transplacental transfer of maternal IgG to the fetus is a specific adaptation that minimizes deficiencies in the production of antibodies (8). Transplacental passage of antibodies *via* maternal immunization can reduce the risk of vaccine-preventable diseases that may occur in early weeks of life in preterm infants (9, 10) (11). For instance, vaccination against influenza, pertussis and pneumococcal infections that have been clinically used (11, 12) Furthermore, use of formulations containing hyperimmune IgG antibodies against specific antigens to treat diseases has been considered. Passive transfer of IgG promotes opsonic activity and antibody dependent cytotoxicity, activates complement, and improves neutrophilic chemotaxis (8, 13, 14). The consensus for passive immunization approaches in preterm neonates has garnered great support (13–17). Vaccine-based approaches for combating candidiasis in neonates could be a viable option because, i) neonatal candidiasis is a lethal disease in a sensitive population, despite antifungal therapy, ii) *C. albicans* antigens are available as targets for immunotherapeutics development, iii) Such approaches have been successful in protection against other diseases in new born infants.

Harnessing immunodominant cell wall proteins of *C. albicans* (Als3p and Hyr1p) for developing immunotherapeutic modalities have proven to be valuable for combating a host of *Candida*-associated infections (18–24). To elaborate, we have previously reported on the efficacy of NDV-3 (the N-terminus of recombinant Als3p formulated with alum) in preventing disseminated candidiasis in mice infected with *C*. *albicans* or with *C. auris (* 19, 25). Vaccination with Als3p coupled with complete/incomplete Freund’s adjuvant also protects mice from hematogenously disseminated candidiasis by other *Candida* species including *C*. *glabrata*, *C*. *tropicalis*, *C. krusei* and *C*. *parapsilosis (* 19, 26). Besides Als3, another cell surface protein Hyr1p [contributes to *C. albicans* virulence by resisting phagocyte killing (27)] has been the subject of investigation by our group and has shown outstanding efficacy against disseminated candidiasis (24, 27). Interestingly, Hyr1p shares striking three-dimensional structural homology with cell surface proteins of Gram-negative bacteria and active vaccination with recombinant N-terminus of Hyr1p (rHyr1p-N) or passive immunization with anti-Hyr1p antibodies protect mice from *Acinetobacter* infection (21, 22). Our prior successes with these novel vaccine candidates in protection against *Candida* infections form a robust premise for their evaluation as immunotherapeutic interventions against candidiasis in neonates. Here, we performed a proof-of-concept study to evaluate if anti-Als3p or anti-Hyr1p antibodies are important for prevention of disseminated candidiasis in neonates. We show that vaccination of mothers with rAls3p-N or rHyr1p-N formulated in CFA/IFA, generates antibodies that transfer efficiently and at high titers into neonates *via* the placenta. These transplacentally transferred antibodies significantly reduce fungal burden in the kidneys of neonatal mice. Moreover, adoptive transfer of sera from vaccinated mothers into pups also affords similar level of protection, including against fluconazole resistant *C. albicans*. Neutrophils were found to be important for the protective outcome. These studies are the first step in the direction of discovering immunotherapeutic strategies for combating life-threatening *Candida* infections in newborns.

## Results

### Anti-rAls3p-N or anti-rHyr1p-N antibodies transfer transplacentally and reduce fungal burden in kidneys of pups

**Figure 1:**
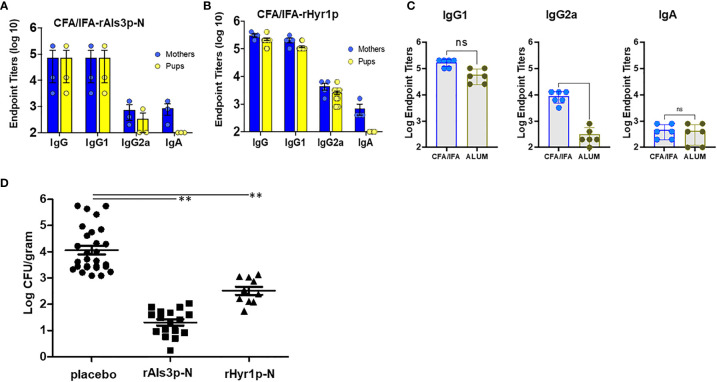
Active vaccination of Female BALB/c mice induce robust antibody titers that cross through placenta to induce protection in their newborn mice against *C. albicans* infection. Female BALB/c mice (6-weeks old) were vaccinated with Als3p-N or Hyr1p-N vaccine adjuvanted with CFA/IFA or adjuvant alone (placebo group) on day 0, 21 and 35. On day 35, mice were mated and kept in cages until the pups were delivered. Sera were obtained on day 6 post-partum both from mothers and their pups for antibody determination **(A-C)**. Als3p-N vaccinated female BALB/c mice **(A)** and Hyr1p-N vaccinated mice **(B)** induce robust total IgG and IgG isotype and IgA antibodies, which efficiently transferred (equivalent titers) to their pups (except IgA). **(C)** CFA/IFA adjuvanted Hyr1p-N vaccine showed similar IgG1 and IgA isotype antibodies, but higher levels of IgG2a in CFA/IFA vaccinated mice. **(D)** Fungal burden in kidneys harvested from 3 day old pups delivered from mothers vaccinated with placebo, CFA/IFA or rAls3p-N or rHyr1p-N + CFA/IFA, three days after infecting with *C. albicans* (3 x 10^7^ cells). **P <0.01 treatment vs. placebo sera vaccinated mice.

We sought to investigate the extent of antibody titers generated in adult female mice vaccinated by CFA/IFA-adjuvanted *C. albicans* antigens. Six to eight week old Balb/c mice were sub-cutaneously (s.c.) vaccinated individually with rAls3p-N (100 µg) or rHyr1p-N (30 µg) formulated in CFA, and then boosted with the same antigens mixed in IFA on day 21 and day 35. Seven days after the second boost, sera from vaccinated and placebo (mice vaccinated with CFA/IFA alone) were collected for determining the anti-rAls3p-N and anti-rHyr1p-N antibodies using ELISA plates coated with rAls3p-N or rHyr1p-N, respectively. Both antigens induced a robust antibody immune response, while no antibodies were found in sera of placebo treated mice. As expected from our previous studies on rAls3p-N (28) and rHyr1p-N (21), IgG1 was the predominant isotype followed by IgG2 and then IgA (**Figures 1A, B**). To understand how these titers compared to alum adjuvant, which we have previously reported in most of our efficacy studies, we compared the IgG isotypes in sera of mothers vaccinated by CFA/IFA-rHyr1p-N and alum-rHyr1p-N. While IgG1 and IgA had similar titers in both adjuvants, IgG2 quantities in CFA/IFA-rHyr1p-N vaccinated mice were a striking 15-fold higher than alum-rHyr1p-N vaccinated mice sera (**Figure 1C**). We next examined if these mice were able to transfer antibodies to their pups. A day before mating, female mice were given the second boost and IgG titers were measured in delivered pups (3 day old). Interestingly, there was an efficient transplacental transfer of both IgG1 and IgG2a isotypes, at titers equivalent to those seen in vaccinated mothers (**Figures 1A, B**). To evaluate the efficacy of these placentally-transferred antibodies against neonatal candidiasis, three-day old pups were infected with *C. albicans* and sacrificed after 72 hrs. Pups with transplacentally acquired anti-rAls3p-N or anti-rHyr1p-N antibodies had at least a 2.5 to 1.5 log reduction (p<0.001) in kidney fungal burden, respectively, compared to pups of mice treated with the placebo (**Figure 1D**).

### Adoptive transfer of sera from vaccinated mothers protect pups from *C. albicans*

**Figure 2:**
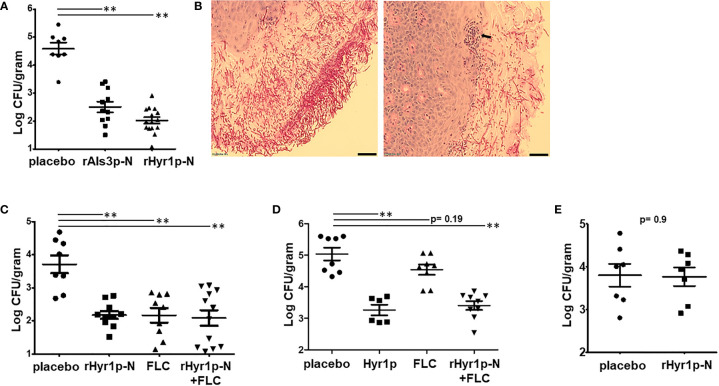
Passive vaccination by rAls3p-N or rHyr1p-N antisera protect pups from candidiasis. **(A)** Fungal burden in kidneys harvested from pups infected with *C. albicans* on day 3 of birth, and treated with sera obtained from mice vaccinated with rAls3p-N, rHyr1p-N or adjuvant alone (placebo). Treatment was on day 0 (4 h post infection) and day 2 relative to infection. **(B)** Histopathology during systemic *C. albicans* infection in pups. Sections stained with PAS stain. Arrows indicate *C. albicans* hyphae invaded kidney tissues. Scale bar = 10 µm. **(C)** Kidney fungal burden in pups infected with a fluconazole (FLC) sensitive strain SC5314 and treated with control placebo sera (n= 8 pups), rHry1p-N anti-sera (n= 10 pups), or FLC alone (n= 9 pups), or a combination of rHyr1p-N+FLC (n= 12 pups). **(D)** Kidney fungal burden in pups infected with a FLC resistant strain CA6 and treated with control placebo sera (n= 8 pups), rHry1p-N antisera (n= 6 pups), FLC alone (n= 8 pups), or a combination of rHyr1p-N + FLC (n= 9 pups). **(E)** Kidney fungal burden in pups depleted of neutrophils, infected with *C. albicans* and treated with either the sera from placebo vaccinated mice (n= 7 pups) or rHyr1p-N anti-sera (n= 7 pups). **P <0.01 treatment vs. placebo sera vaccinated mice.

To further confirm the potential of anti-rHyr1p-N and anti-rAls3p-N antibodies in protecting mice from *C. albicans* infection, 3-day old pups were treated with sera collected from their respective vaccinated (or placebo) mothers 2 h post infection and repeated on day 2 prior to sacrificing them on day 3 to recover their kidneys. Indeed, passive vaccination of pups reduced fungal burden in the kidneys by >2 logs, compared to placebo-sera treated pups (p<0.001; **Figure 2A**). We sectioned the kidneys of pups treated with sera from placebo treated or CFA/IFA-rHyr1p-N vaccinated mothers for histopathology. Extensive infection was discovered in kidneys of control pups with *C. albicans* hyphal cells penetrating the kidney cortex (**Figure 2B**). In contrast, adoptively vaccinated pups displayed strikingly low fungal burden and recruitment of neutrophils at the site of infection. We next tested the potential of rHyr1p-N antisera in synergizing with fluconazole in enhancing the efficacy of this antifungal against neonatal candidiasis. Both the rHyr1p-N antibodies containing sera and fluconazole reduced kidney fungal burden by ~1.5 log when used as monotherapy (p<0.001; **Figure 2C**). Although the combination treatment of fluconazole and rHyr1p-N antibodies had similar reduction in tissue fungal burden (*i.e.*, 1.5 log), 40% of the mice were completely protected from infection (fungal burden at the limit of detection), compared to 10% in fluconazole- and 0% in anti-rHyr1p-N antibodies-treated mice. Interestingly and highly promising, the anti-rHyr1p-N antibodies significantly protected mice infected by a fluconazole resistant strain when used alone or in combination with fluconazole. Specifically, compared to the placebo treated pups, antisera containing anti-rHyr1p-N antibodies alone or in combination with fluconazole reduced kidney fungal burden by two logs (p<0.001), while fluconazole alone resulted in an insignificant reduction of < 0.5 log (p=0.19) (**Figure 2D**).

### Neutrophils are important for protection against neonatal candidiasis

We have reported previously that Hyr1p confers resistance to innate immune cells, and sera containing anti-rHyr1p-N antibodies can enhance mouse neutrophil killing activity by directly neutralizing rHyr1p effects (24, 27). Thus, we inspected the role of neutrophils in the presence of anti-rHyr1p-N antibody-mediated protection against *C. albicans* infection in neonatal mice. Briefly, peripheral neutrophils in naïve newborn mice (2 d old) were depleted by injecting anti-Ly-6 antibodies (20 μg/gram body weight) intraperitoneally. Induction of neutropenia was verified in a sub-set of mice 24 h later (on the day of infection) by staining splenocytes for neutrophil phenotypic markers (Cd11b and Ly6) and analysis by flow cytometry. The mice administered with anti-Ly-6 antibodies showed abrogation of Cd11b+Ly6+ cell population in the splenocytes, while control isotype antibody maintained significant frequency of this population (**S1**). The infected neutropenic neonate mice were treated with anti-Hyr1p-N antibodies (or placebo) containing sera on day +1 and day +2, relative to infection. The protective effect of anti-rHyr1p-N antibodies against candidiasis was abrogated in the absence of neutrophils, wherein the kidney fungal burden between control *vs.* anti-rHyr1p-N sera was not significantly different (p=0.9; **Figure 2E**). These results are in agreement with our previous observations in adult mice, and highlight that neutrophils are required for the anti-Hyr1p-mediated protection against *C. albicans* infection.

## Discussion

Neonatal candidiasis is associated with significant morbidity and mortality in infants. Few clinical signs or laboratory assays have been validated for candidiasis in infants, and successful outcomes rely heavily on early removal of indwelling catheters, followed by prompt treatment by standard of care antifungal drugs such as fluconazole (29). Despite antifungal treatment, one in five very low birthweight neonates will succumb to candidiasis; one in two of the survivors will demonstrate severe neurodevelopmental impairment (30, 31) and suffer end-organ damage in the central nervous system, heart, and genitourinary tract (30). Clearly, alternative strategies to combat life threatening candidiasis in an immunocompromised population such as premature infants are sorely needed.

We performed a proof-of-concept study to evaluate if antibodies raised against *Candida* antigens have the capability of protecting against neonatal candidiasis. Previous studies from our group have shown that vaccine candidates based on *C. albicans* cell surface proteins such as rAls3p-N and rHyr1p-N, adjuvanted with alum, can protect against candidiasis in several animal models (19, 20, 27, 32). Furthermore, anti-rHyr1p-N antibodies have robust cross-kingdom efficacies extending to protection of mice from lethal infections also by Gram-negative bacteria (21, 22). Thus, expansion of the scope of these two antigens, for protection of neonates against disseminated *Candida* infections is the most logical step, in light of the high mortality rates despite antifungal treatments in VLBW infants.

The adaptive immune response in newborns is underdeveloped, having had no previous exposure to microbes *in utero*, and due to the impaired functions of B and T cells (33). Protection against infection in a premature neonate essentially depends upon the innate immune system, and on antibodies acquired passively from the mother (7). We found that, vaccinating female mice with rAls3p-N or rHyr1p-N adjuvanted with CFA/IFA produced a robust antibody response with titers as high as 5 logs. CFA/IFA are considered as the most effective adjuvant available for consistently producing high titer antibodies to diverse antigens, and are irreplaceable and vital for immunology research and antibody production. Importantly, we found that the quality of these antibodies were predictors of efficacy against *Candida* infections. In particular, IgG2 titers generated by CFA/IFA vaccination was 15-fold higher than that produced by alum. We have previously reported that IgG2 titers are biomarkers of efficacy for protection of women from recurrent vulvovaginal candidiasis (28). Our hypothesis was that in the absence of adaptive immunity, antibodies are important for protection from neonatal candidiasis. The adjuvants CFA/IFA generated a high IgG2 response prompting us to perform all our studies using these adjuvants.

Vaccination of mothers by CFA/IFA-rAls3p-N or CFA/IFA-rHyr1p-N, before conception, generated antibodies that robustly transferred *via* the placenta to the neonates, and accumulated in the pups at concentrations comparable to titers present in their mothers. In humans, IgG transfer from mother to fetus begins as early as 13 weeks of gestation, with a continuous rise in IgG levels in fetal circulation between 17 and 41 weeks of gestation. Fetal IgG concentrations reach 50% of the maternal concentrations at weeks 28–32, and increase rapidly thereafter (34, 35). Thus, presence of high titer antibodies in full-term delivered pups was not surprising. Furthermore, IgA is incapable of transplacental passage and hence the titers of IgA in pups was negligible. Post rAls3p-N and rHyr1p-N vaccination of mothers, the placentally transferred antibodies reduced kidney infection in pups by a 1.5 to 2 logs compared to placebo (CFA/IFA with antigen) vaccinated mice. Maternal immunization can reduce the risk of vaccine-preventable diseases that may occur in the early weeks of life, especially in the pre-term infants (11). One of the best examples is vaccination against influenza that can protect pregnant women from a life threatening disease, induce protective specific antibody levels and protect infants in the first weeks of life (11). Other examples are vaccinations against pertussis and pneumococcal infections (11, 12).

Our studies have some limitations. First, experiments were performed in full term pups, despite the fact that neonatal candidiasis is predominant in low birth weight preterm infants. However, it is impossible to keep prematurely delivered pups alive for longer than a few hours. Besides, disseminated candidiasis is a serious problem also in intubated full-term infants, whereas mucosal candidiasis in the form of oral thrush is a frequent occurrence in term babies. Hence, we do not consider this as a debilitating weakness. Second, despite several attempts, we were unsuccessful in establishing a survival model of disseminated infection in pups; hence, we performed all our efficacy studies focusing on recording changes in fungal burden in the primary target organ (kidneys), an endpoint that has been used in numerous publications as a parameter for evaluating therapeutic efficacies of antifungal drugs and vaccines alike (36–40). Third, the route of adoptive transfer in neonates was intraperitoneal (i.p), rather than intramuscular that has been the preferred route in our previous preclinical investigations and in clinical trials (18, 20, 32). The anatomy of pups makes i.p the most relevant route to evaluate efficacy of transferred antibodies, and has been extensively reported in the area of neonatal vaccinology (41, 42). In fact, i.p administration of vaccines has long been used and studied as an experimental immunization route for the induction of systemic and mucosal immunity in animal models of vaccination (43).

Adoptive transfer of antisera collected from rAls3p-N or rHyr1p-N vaccinated mice, into three-day old pups, significantly protected the infants from candidiasis by >1- to 1.5-fold, versus placebo vaccinated pups (antisera of mice treated with CFA/IFA alone). Administration of passive antibodies to protect from neonatal infections has shown tremendous promise. Passive transfer of IgG promotes opsonic activity and antibody dependent cytotoxicity, activates complement, and improves neutrophilic chemotaxis (44). Case reports from several clinical trials have indicated that antenatal administration of anti-CMV IgG may be associated with significantly favorable outcomes (a rate of 16:1) in fetuses suspected of having congenital CMV infection (45–49). Such encouraging success stories warrant further development of passive antibody transfer approaches for protection of VLBW neonates against disseminated candidiasis, the incidence of which is several fold higher than CMV.

Adoptive transfer of CFA/IFA-rHyr1p-N antisera however was not as effective in neutrophil-depleted mice. Neutrophils are a major component of the innate immune system, and the adaptive immune response in pups is absent. Thus, loss of neutrophils had a significant impact on the protective nature of the anti-rHyr1p-N antisera. We have shown previously that anti-rHyr1p-N polyclonal antibodies enhance mouse neutrophil killing activity by directly neutralizing rHyr1p effects *in vitro*, and that neutrophils are important for protection post vaccination by Hyr1 (21, 27).

Immunotherapies are not expected to replace antibiotics, rather serve as adjunct therapies to enhance the potency of drugs. We have previously shown that anti-Hyr1 antisera synergizes with antibiotics for prevention of the Gram-negative bacteria *Acinetobacter baumannii* growth *in vitro (* 21 *)*. Thus, we evaluated the potential of the CFA/IFA-rHyr1p-N sera to synergize with fluconazole, in protecting mice from candidiasis. Reassuringly, the antisera was not antagonistic when used in combination with fluconazole and could significantly abrogate infection by fluconazole resistant strains of *Candida*, indicating that passive vaccination strategy using anti-rHyr1p-N antibodies could be an important strategy to strengthen therapeutic attempts to combat candidiasis in neonates.

Ours is the first step in the direction of discovering immunotherapeutic strategies for combating life-threatening *Candida* infections in newborns. Our findings collectively highlight the potential of using rAls3p-N and/or rHyr1p-N based maternal vaccine strategies, or antibody-based immunotherapies, for protection from debilitating and lethal candidiasis in neonates. In future, FDA-approved commercially available adjuvants with profiles of antibody isotypes similar to CFA/IFA could be used in conjunction with the antigens for protection against neonatal candidiasis.

## Methods

### Strains and media conditions

*C. albicans* SC5314 is a well-characterized strain and was the source of the N-terminus of Als3p used to develop both the NDV-3A and the Hyr1p-N vaccines (18, 21). Fluconazole resistant *C. albicans* strain CA6 was received from the Fungus Testing Laboratory at the University of Texas Health Science Center at San Antonio. The MIC_50_ of fluconazole against this strain is 16 (50). Routinely, the organisms were cultured overnight in yeast peptone dextrose (YPD) broth (Difco) at 30°C with shaking prior to use for *in vitro* and *in vivo* assays. In all studies, *C. albicans* yeast cells were washed twice with endotoxin-free Dulbecco’s phosphate-buffered saline (PBS), suspended in PBS and counted with a hemocytometer to prepare the final inoculum.

### Neonatal mouse model and vaccination strategy

All mice procedures were approved by the IACUC of The Lundquist Institute at Harbor-UCLA Medical Center, according to the NIH guidelines for animal housing and care. Ten to 12 week old BALB/c mice (depending upon active or passive vaccination studies) were allowed to mate at a 1:1 ratio of male to female, and pups delivered ~19-21 days post-conception. Post-partum day 3 pups were weighed and randomized within cages prior to infection with 50 µl of 3 × 10^7^ C*. albicans* yeast cells. Infection was performed i.p with 0.5 ml Kendall Monoject™ Safety Syringe with 29½ G needle (Tyco Healthcare, Cat # 8881511136). Pups were examined daily, and at indicated time-points were humanely euthanized for the analysis of fungal burden and histology.

For tissue fungal burden, kidneys were harvested, homogenized in sterile PBS, and fungal burden enumerated by quantitative culturing of colony forming units (CFU). The CFUs were counted after 48 h of incubation at 30°C and expressed as CFU/g tissue.

For histology, whole kidneys of pups were fixed and kept in 4% buffered formalin until ready for processing. Fixed kidneys were sectioned and stained with periodic acid-Schiff (PAS) using conventional staining methods. Tissue sections were visualized and imaged using a bright field microscope.

For active vaccination studies, 6 weeks old female BALB/c were vaccinated on Day 0 with Als3p or Hyr1p antigens (recombinant Als3p-N [100 µg] or Hyr1p-N [30 µg]) mixed with complete-Freund’s adjuvant (CFA). Mice were boosted twice with the same doses of antigens mixed with incomplete-Freund’s adjuvant (IFA) on day 21 and again on day 35 prior to mating them on day 36. Our work with adult mouse models have demonstrated that two boost is more protective compared to one boost against candidemia. Since the goal was to generate the best antibody response to test in neonatal candidiasis, we chose to go with two booster doses. Adjuvant alone served as placebo control group. Delivered neonatal pups were infected and sacrificed as described above for fungal burden. The vaccinated female BALB/c mice were bled to generate sera for use in passive vaccination studies.

For passive vaccination studies, pups delivered from naïve mice were infected with *C. albicans* 3 days post-partum. Two to three hours later pups were treated IP with 50 µl of pooled anti-Als3p-N, anti-Hyr1p-N or control sera (previously collected from vaccinated adult female BALB/c mice, see below for details) by using 0.5 ml Kendall Monoject™ Safety Syringe with 29½ G needle (Tyco Healthcare, Cat # 8881511136). Pups were treated again with the same volume two days after the first treatment, and sacrificed one day later (day 6 old) for kidney fungal burden and histology.

For combination treatment with antisera and fluconazole, pups were treated with antisera (after 2-3 hr and at 48 h of infection, as described above). For fluconazole treatment, pups were administered with 50 µl of the drug at 5 mg/kg daily, starting 24 h after infection (i.e., at 24 h and 48 h post infection). Pups were sacrificed on the 3rd day after infection.

### Measurement of antibody titers

To determine the anti-rAls3p-N and rHyr1p-N specific antibodies (total IgG and other isotypes) in Als3p-N or Hyr1-N (mixed with CFA/IFA) vaccinated mice, 96-well plates were coated with 5 µg/ml of recombinant Als3p or Hyr1p in bicarbonate/carbonate coating buffer (pH 9.6) overnight at 4°C. The next day, the plates were washed three times with 1x wash buffer (PBS containing 0.05% tween-20) and blocked with 3% BSA solution for 2 hours at room temperature. After washing three times, 2-fold serially diluted serum samples were added to the plates in duplicates and incubated for two hours. In some ELISA, we also used historic samples from mice vaccinated with Hyr1p-N mixed with Alum adjuvant. After incubation, the plates were washed three times and 1:1000 diluted anti-mouse IgG (Jackson, Cat#115-035-164), IgG1 (eBioscience, Cat#18-4015-82), IgG2a (Invitrogen, Cat#61-0220) or IgA (Southern Biotech, Cat#1040-05antibodies labelled with peroxidase were added and incubated for 1 hour at room temperature. Finally, the plates were washed five times with washing buffer, TMB (3,3′,5,5′-Tetramethylbenzidine) substrate (Invitrogen, Cat#00-4201-56) was added. Color development was allowed for 5–10 minutes and the optical density was measured at 450 nm (OD450) after the reaction was stopped with 1 N sulfuric acid (Sigma, Cat#339741). The measured OD450 nm values were converted to antibody titers by the reciprocal of the highest dilution factor that gives OD450 above the cut-off value (Average OD450 of blank wells + 2 standard deviation).

### Neutrophil depletion in neonate mice

To decipher the role of neutrophils in antibody-mediated protection of neonates mice against *C. albicans* infection, we depleted the peripheral neutrophils before the infection. Briefly, 2 days old naïve newborn mice were injected with 50 µl (20 µg/gram body weight) of InVivoPlus anti-murine Ly-6 antibodies (BioXcell, Clone 1A8, Cat# BP0075-1-R005) or isotype-matched antibodies (BioXcell, Clone 2A3, Cat# BP0089-R007) IP. This treatment specifically depletes neutrophils expressing Ly-6 marker on their surface. Twenty-four hours after the treatment (on the day of infection), neutrophil depletion was verified in a sub-set of mice by staining 1 million splenocytes/mouse for neutrophil phenotypic markers using anti-mouse Cd11b (Rat anti-Mouse, BB700, Clone: M1/70, BD Horizon, Cat #BDB566416) and anti-mouse Ly6 (Rat anti-Mouse, FITC, Clone: RB6-8C5, BD, Cat #BDB553127) and acquiring data by flow cytometry. Absence of Cd11b+Ly6+ population in spleen confirmed neutrophil depletion.

### Statistical Analysis

Experiments were performed at least twice with at least 4 mice/group/experiment. GraphPad Prism 9 software was used to analyze the results. Data presented as means with standard errors of the means (SEM). Differences in fungal burden were determined using the non-parametric Mann–Whitney U-test. Differences between groups were considered significant at p-values of <0.05.

## Data Availability Statement

The raw data supporting the conclusions of this article will be made available by the authors, without undue reservation.

## Ethics Statement

All mice procedures were approved by the IACUC of The Lundquist Institute at Harbor-UCLA Medical Center, according to the NIH guidelines for animal housing and care.

## Author Contributions

SS: Designing, implementation, troubleshooting, editing and writing parts of the manuscript; SN: Implementation, data analysis, editing manuscript; AB: Contributed to carry out *in vitro* ELISA assays; AI: Designing, providing proprietary antigens, discussions, writing and editing manuscript to final completion; PU: Conceptualization, designing, implementation, supervision, writing the manuscript. All authors contributed to the article and approved the submitted version.

## Funding

We thank the funding support from NIH that helped enable our studies for this manuscript: NICHD R21HD097480 and NIAID R01AI141794 awarded to PU; NIAID 1R01AI141202-01 awarded to AI.

## Conflict of Interest

The authors declare that the research was conducted in the absence of any commercial or financial relationships that could be construed as a potential conflict of interest.

## Publisher’s Note

All claims expressed in this article are solely those of the authors and do not necessarily represent those of their affiliated organizations, or those of the publisher, the editors and the reviewers. Any product that may be evaluated in this article, or claim that may be made by its manufacturer, is not guaranteed or endorsed by the publisher.

## Supplementary Material

S1 Neutrophil depletion verification in neonate mice. Neonate mice (n=3 pups/group) were treated with isotype-matched control antibodies for the control depletion (top row) or anti-murine Ly-6 antibodies for neutrophil depletion (second row). After 24 hours of treatment, pups were euthanized and spherocytes were collected, homogenized, and counted. One million solenocytes from each pup were used to stain the cells for neutrophil surface markers using anti-mouse CD11b-BB700 and anti-mouse Ly-6 FITC fluorescent antibodies. Splenocytes from control depletion mice were also stained with isotype matched control antibodies (bottom zebra-plot). Depletion group show drastic decrease in Cd11b+Ly-6+ population, whereas this population is very prominent in control depletion group.
