# Supplementary figures and images for: Antibodies targeting Candida albicans Als3 and Hyr1 antigens protect neonatal mice from candidiasis
Source: Front Immunol. 2022 Jul 22;13:925821. doi: 10.3389/fimmu.2022.925821 (PMC9355692; doi:10.3389/fimmu.2022.925821)

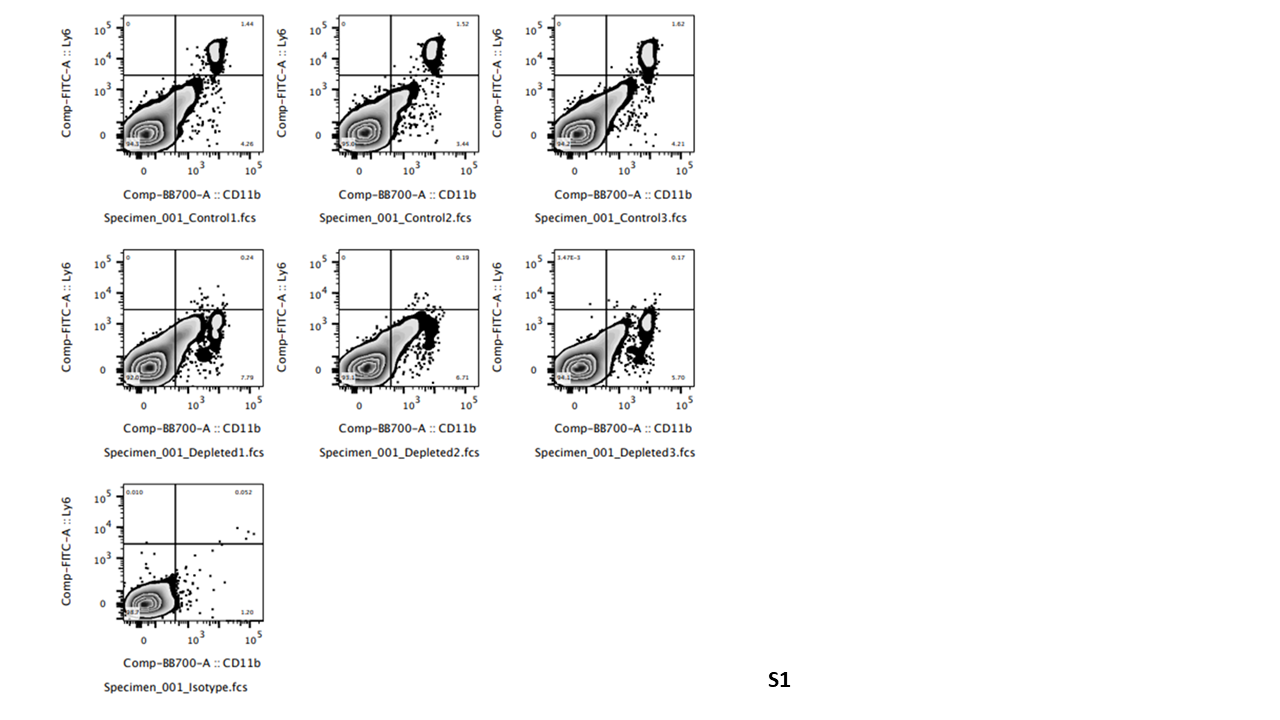

Supplement: S1 — Neutrophil depletion verification in neonate mice. Neonate mice (n=3 pups/group) were treated with isotype-matched control antibodies for the control depletion (top row) or anti-murine Ly-6 antibodies for neutrophil depletion (second row). After 24 hours of treatment, pups were euthanized and spherocytes were collected, homogenized, and counted. One million solenocytes from each pup were used to stain the cells for neutrophil surface markers using anti-mouse CD11b-BB700 and anti-mouse Ly-6 FITC fluorescent antibodies. Splenocytes from control depletion mice were also stained with isotype matched control antibodies (bottom zebra-plot). Depletion group show drastic decrease in Cd11b+Ly-6+ population, whereas this population is very prominent in control depletion group. [file Image_1.tif]
